# Effect of Pore Size on Cell Behavior Using Melt Electrowritten Scaffolds

**DOI:** 10.3389/fbioe.2021.629270

**Published:** 2021-07-02

**Authors:** Yu Han, Meifei Lian, Qiang Wu, Zhiguang Qiao, Binbin Sun, Kerong Dai

**Affiliations:** ^1^Department of Orthopaedic Surgery, Shanghai Key Laboratory of Orthopaedic Implants, Shanghai Ninth People’s Hospital, Shanghai Jiao Tong University School of Medicine, Shanghai, China; ^2^Clinical and Translational Research Center for 3D Printing Technology, Shanghai Ninth People’s Hospital, Shanghai Jiao Tong University School of Medicine, Shanghai, China; ^3^Department of Prosthodontics, Shanghai Ninth People’s Hospital, College of Stomatology, Shanghai Jiao Tong University School of Medicine, National Clinical Research Center for Oral Diseases, Shanghai Key Laboratory of Stomatology & Shanghai Research Institute of Stomatology, Shanghai, China

**Keywords:** melt electrowritten, pore size, scaffold, cell, PCL

## Abstract

Tissue engineering technology has made major advances with respect to the repair of injured tissues, for which scaffolds and cells are key factors. However, there are still some issues with respect to the relationship between scaffold and cell growth parameters, especially that between the pore size and cells. In this study, we prepared scaffolds with different pore sizes by melt electrowritten (MEW) and used bone marrow mensenchymal stem cells (BMSCs), chondrocytes (CCs), and tendon stem cells (TCs) to study the effect of the scaffold pore size on cell adhesion, proliferation, and differentiation. It was evident that different cells demonstrated different adhesion and proliferation rates on the scaffold. Furthermore, different cell types showed differential preferences for scaffold pore sizes, as evidenced by variations in cell viability. The pore size also affected the differentiation and gene expression pattern of cells. Among the tested cells, BMSCs exhibited the greatest viability on the 200-μm-pore-size scaffold, CCs on the 200- and 100-μm scaffold, and TCs on the 300-μm scaffold. The scaffolds with 100- and 200-μm pore sizes induced a significantly higher proliferation, chondrogenic gene expression, and cartilage-like matrix deposition after *in vitro* culture relative to the scaffolds with smaller or large pore sizes (especially 50 and 400 μm). Taken together, these results show that the architecture of 10 layers of MEW scaffolds for different tissues should be different and that the pore size is critical for the development of advanced tissue engineering strategies for tissue repair.

## Introduction

In recent years, tissue engineering technology has made tremendous contributions to tissue regeneration (Guo et al., 2018; Cunniffe et al., 2019; Ruvinov et al., 2019). Tissue engineering technology simulates the regeneration of tissues and organs by combining elements such as biological materials, cells, and biologically active molecules to mimic the structure and function of native tissues and organs (Kang et al., 2018; Kumai et al., 2019). These studies reveal that tissue engineering scaffolds are vital components, whose composition and structure affect the proliferation, differentiation, and gene expression in cells (Mannoor et al., 2013; Li et al., 2017; Schon et al., 2017; Wang et al., 2018).

In past studies, researchers have gradually found that, although tissue scaffolds made by general manufacturing techniques can alter the extracellular environment, the changes are more uniform and largely uncontrollable (Teimouri et al., 2015; Zhang et al., 2017; Ponce et al., 2018; Li et al., 2019). However, the complex environment in normal tissues is significantly different. While studies have investigated the heterogeneity associated with cellular complexity, the heterogeneity of the natural extracellular environment has not been replicated. This is primarily due to the difficulty of creating a 3D environment. Although we have developed many intricate methods to generate complex 2D models or prototypes with mechanical or chemical gradients, these culture conditions may not apply to many cell types (Karageorgiou and Kaplan, 2005; Tanaka et al., 2010; Panadero et al., 2016). It is difficult to achieve 3D effects with 2D models (Warnecke et al., 2018). The development of 3D printing technology has provided a new method for the production of tissue engineering scaffolds (Hung et al., 2016; Daly et al., 2017, 2018; Kosik-Kozioł et al., 2017; Chen et al., 2018). As an advantage, 3D printing technology can control the shape and size of the scaffold during production and even print different structures in different parts. However, the specific effects of the micro-architecture on cells, especially that of the scaffold pore size, are not completely clear.

Therefore, for tissue scaffolds obtained by printing, a better understanding of the effect of pore size on adhesion, proliferation, and expression is needed. The pores of scaffolds can provide space for cell growth, and the scaffold itself provides support for cell adhesion. However, while the scaffold with small pores provide more adhesion support, they inevitably reduce the cell growth space. Furthermore, pore size can affect cell differentiation and gene expression (Matsiko et al., 2015). Therefore, a balance between the two is crucial (Zhang et al., 2016). The effect of scaffold pores on cell behavior may be due to specific cell–scaffold interactions. In particular, some studies have shown that cell morphology plays an important role in cell differentiation, which is also associated with the scaffolds (Glaeser et al., 2010; Abdeen et al., 2014; Teixeira et al., 2016; Li et al., 2020).

Therefore, it is important to study and understand the mechanisms underlying scaffold–cell interactions and the subsequent cell differentiation. Previous studies have investigated the effects of scaffold pores on cells but reported conflicting findings. For example, some studies suggested that pores were suitable for chondrogenic differentiation at 20–150 μm (Nehrer et al., 1997; Stenhamre et al., 2011), while others suggested that 250–500 μm was suitable for chondrocyte proliferation and gene expression (Griffon et al., 2006; Lien et al., 2009; Nelson et al., 2021). Besides this, different cells have different sizes and shapes so that the optimal pore size may differ for different cell types. Some studies suggested that the optimal pore size should be about 30 times the size of the cell itself (Salem et al., 2002; Oh et al., 2010; Matsiko et al., 2015). Therefore, this study would examine the effects of pore size on the proliferation and differentiation of different cells.

**FIGURE 1:**
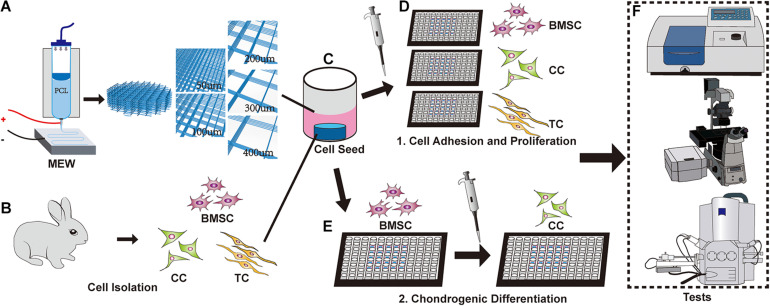
Schematic of the experimental design. **(A)** Melt electrowritten (MEW) printer. The upper part is barreled. The needle is connected to the lower receiving plate to apply an electric field. The pore size of the scaffolds is controlled to 50, 100, 200, 300, and 400 μm, respectively. **(B)** New Zealand white rabbits were used to obtain bone marrow mesenchymal stem cells (BMSCs), chondrocytes, and tendon cells. **(C)** Implantation of different cells on the MEW scaffolds. **(D)** Cultured cells on a scaffold. **(E)** Induction of chondrogenic differentiation of BMSCs on scaffolds. **(F)** Fluorescence staining, microscopy, CCK-8, and other relevant tests were performed.

To avoid the effects of other aspects of the scaffold, polycaprolactone (PCL) was chosen by virtue of its excellent thermoplasticity and biocompatibility (Felice et al., 2018; Bahcecioglu et al., 2019). Moreover, PCL has excellent stability as a degradable material and can be mixed with many metals, drugs, biological factors, etc. (Ghorbani et al., 2015). At present, PCL is widely used for printing various organizational structures and is one of the most commonly used materials (Pok et al., 2013; Kundu et al., 2015; Hanas et al., 2016). In this study, we hypothesized that smaller-pore scaffolds provided more adhesion support to the cells, thereby improving cell adhesion, while larger pores provided more space for cells to proliferate, thereby avoiding premature contact inhibition (Woodfield et al., 2005). To verify this, we used melt electrowritten (MEW) printing scaffolds (Hrynevich et al., 2018; Brennan et al., 2019; Youssef et al., 2019). Although humans have large-sized tissues and organs, the size of the smallest functional units of the tissues and organs is often in the micrometer range, potentially giving rise to challenges associated with conducting related research. MEW printing has advantages such as high precision (Castilho et al., 2019; Ye et al., 2019). This technique includes an electric field based on ordinary melt-extrusion printing, which can better control the printing accuracy and increase the porosity (Daly et al., 2017). The printed fiber diameter is about 10 μm, which can give the cell enough mechanical support while avoiding interference from other factors (Zhu et al., 2017; Zamani et al., 2018; Ye et al., 2019; Youssef et al., 2019) (Figure 1).

## Materials and Methods

### Materials

The following reagents were used in this study: PCL (average molecular weight Mn = 45.00 kDa; Sigma-Aldrich, CA, United States), a-DMEM, DMEM/F12 (HyClone, UT, United States), fetal bovine serum (FBS; Gibco, NY, United States), TRITC and FITC phalloidin (R415 and A12379, Invitrogen, CA, United States), 4,6-diamidino-2-phenylindole (DAPI, C0060, MaoKang, Shanghai, China), cell counting kit-8 (CCK-8; Dojindo, Kumamoto, Japan), primary antibodies anti-collagen type II (COL II; ab185430), anti-collagen type I (COL I; ab6308), anti-aggrecan (AGC; ab3778), SOX-9 (SOX9; ab76997; Abcam, Cambridge, United Kingdom), Alexa Fluor 555-conjugated anti-mouse antibody (A32727, Invitrogen, CA, United States), RNeasy mini kit (Qiagen, Hilden, Germany), SuperScript^TM^ III Reverse Transcriptase (Bimake, Shanghai, China), and Sirius red solution, Safranin, and fix-green solution (YifanBio, Shanghai, China).

### Fabrication of the Scaffold

In this study, PCL was used as the scaffold material that was placed in the charging barrel. The MEW 3D printer (TL-Direct Writing 150) was started, and the barrel temperature was adjusted to 65°C. The distance between the needle and the printing table was about 3 mm, and the printing station had a negative electrical charge. Furthermore, the printing voltage changed as the printing height changed (6 kV, voltage; 1,500 mm/min, printing speed; 22°C, ambient temperature; 35%, humidity). Additionally, the print needle model was 21G, with 100 kPa of air pressure. The conditions remained stable during printing. Different pore supports were fabricated according to the drawing model, and each scaffold was 200 μm in thickness (10 layers).

### Cell Isolation and Culture

Three-week-old New Zealand white rabbits were euthanized to extract bone marrow mesenchymal stem cells (BMSCs), chondrocytes (CCs), and tendon stem cells (TCs). For BMSC isolation, both ends of the femur and tibia were removed. Subsequently, 10 ml of high glucose α-DMEM medium containing 10% FBS, 1% penicillin/streptomycin, and 100 U/ml heparin sodium was used to repeatedly rinse the bone marrow cavity and elute the bone marrow. Furthermore, stem cells were collected in a centrifuge tube and centrifuged at 100 × *g* for 5 min. The supernatant was discarded, and the pellet was resuspended in high-glucose α-DMEM medium with 10% FBS and 100 U/ml penicillin/streptomycin. Finally, the cells were incubated in an incubator at 37°C and with 5% CO_2_, and the medium was changed every 2–3 days.

For CC isolation, the femur and tibia were removed, and a scalpel and scissors were used to separate the articular cartilage and cut the cartilage into pieces (<0.1 mm). These pieces were suspended in 10 ml DMEM/F12 medium containing 1% collagenase II (2275MG100, Biofroxx, Germany) and mixed. The suspension was then placed in a shaker with 37°C constant temperature and digested at 100 r/min for 2 h. Furthermore, the mixture was centrifuged at 100 × *g* for 5 min, followed by the removal of the supernatant, and cultured in DMEM/F12 medium containing 10% FBS, 50 μg/ml ascorbic acid, and 100 U/ml of penicillin/streptomycin in an incubator at 37°C with 5% CO_2_. The medium was changed every 2–3 days.

For TC isolation, the Achilles tendon was removed and cut into pieces (<0.1 mm). Subsequently, the tendon pieces were suspended in 10 ml DMEM/F12 medium containing 1% collagenase I (1904MG100, Biofroxx, Germany), blown, and mixed. The suspension was placed in a constant temperature shaker at 37°C and digested at 100 r/min for 2 h. The mixture was centrifuged at 100 × *g* for 5 min, and the supernatant was discarded. Finally, the cells were cultured in DMEM/F12 medium containing 10% FBS and 100 U/ml of penicillin/streptomycin in an incubator at 37°C with 5% CO_2_. The medium was changed every 2–3 days.

The cells were observed daily. The extracted primary cells were marked as P0, and the P2 phase cells were used in the experiments.

### Cell Seeding and Culture on Scaffolds

After the scaffolds were printed, they were immersed in 75% alcohol and placed in an ultraviolet disinfection box to be disinfected for about 12 h. After removal, the scaffolds were rinsed with PBS three times (5 min). The scaffolds were then placed in six-well plates or a 96-well plate. Then, P2 BMSCs, CCs, and TCs were adjusted to a density of 3,000 cells/ml; 1 ml of cell suspension/well was added to six-well plates, and 100 μl of cell suspension/well was added to 96-well plates. After a 4-h incubation, the six-well plates were supplemented with 1 ml of medium per well, and the 96-well plates were supplemented with 100 μl of medium per well.

### Morphological Characterization of Scaffolds by Scanning Electron Microscope

The pore size and fiber diameter of the scaffold were printed using a pre-designed model. The printed scaffold was mounted on aluminum foil and sprayed with 6-nm-thick platinum (OPC-80T, SPI Supplies, West Chester, PA, United States). The sample with cells was fixed using glutaraldehyde for 4 h and then fixed with citric acid for 2 h. After fixation, it was dehydrated using ethanol (50, 70, 85, 90, and 100%) and then placed in a lyophilizer (Free Zone, Labconco, United States) overnight. The dried sample was coated with platinum. After coating with a 6-nm-thick layer of platinum, scanning electron microscopy (Leica, Germany) was used to observe the scaffold.

### Fluorescence Staining and Microscopy

Scaffolds with cells were fixed using 4% paraformaldehyde. After 1 h, the specimens were removed, washed with PBS three times for 5 min, immersed in Triton X-100 (0.1%) for 5 min, and washed three times with PBS (5 min each). Furthermore, the specimens were incubated with FITC-labeled phalloidin for 45 min at 21°C and then washed with PBS three times (5 min each). Next, the specimens were incubated with DAPI for 10 min at room temperature and then washed with PBS three times (5 min each). Finally, fluorescence microscopy (Leica, Germany) was used to observe the cells after staining.

### Cell Counting Kit-8 Test

The CCK-8 test was used to measure the adhesion and proliferation of different cells in various pore scaffolds. After the scaffolds were placed in a 96-well plate, BMSCs, CCs, and TCs were seeded on each of the pore scaffolds. At 4, 7, 14, and 21 days, the cell scaffolds were transferred to another 96-well plate. Then, 10% CCK-8 reagent was added to each well and placed in an incubator at 37°C for 1 h in the dark. Subsequently, 100 μl of each well medium was transferred to a clean 96-well plate. The absorbance of the culture media was measured with a microplate reader (Thermo Fisher Scientific, Inc.) at 450 nm (630 nm as a reference, *n* = 4).

### Cell Seeding and Chondrogenic Differentiation on Scaffolds

P2 BMSCs were used to study chondrogenesis, 1 ml of cell suspension (3,000 cells/ml) was added to each well of the six-well plate containing scaffolds. After the cells adhered to the scaffold, the medium was replaced with chondrogenic differentiation medium (DMEM, 1% insulin–transferrin–selenium, 100 μg/ml sodium pyruvate, 40 μg/ml proline, 10^–7^ M dexamethasone, 50 μg/ml ascorbic acid, and 10 ng/ml TGF-β1). The cell condition was closely observed, and the medium was changed every 2 days.

### Histological Staining

After 21 days, the specimen were removed, washed two to three times with PBS, and fixed with 4% paraformaldehyde for 1 h. Sirius red, Safranine O, and Fast-Green staining were performed separately. For Sirius red staining, the sample was immersed in PBS and washed. Then, the specimens were stained with Sirius red for 10 min, washed with ethanol, and observed under the stereoscope. For Safranine O and Fast-Green staining, the sample was immersed in PBS and washed. Subsequently, Fast-Green was added for 15 min and washed with PBS, followed by incubation with red dye solution for 5 min and treatment with color separation solution for 20 s. After PBS washing, the specimens were observed under a stereoscope.

### Immunofluorescence and Confocal Microscopy

The scaffolds with cells were fixed with 4% paraformaldehyde. After 1 h, the specimens were removed, washed with PBS three times for 5 min, immersed in Triton-X (0.1%) for 5 min, and washed three times with PBS for 5 min each. The specimens were blocked with BSA for 1 h and primary antibody (COL II, COL I, AGC, and SOX9; diluted according to the manufacturer’s protocol) for 12 h at 4°C and then washed with PBS three times for 5 min each. Subsequently, they were incubated with the Alexa Fluor 555-conjugated anti-mouse antibody for 1 h at room temperature, washed three times with PBS (5 min each), and then incubated with phalloidin and DAPI as described above in fluorescence staining and microscopy. The specimens were observed by confocal microscopy (Leica, Germany) after staining. ImageJ (National Institutes of Health, Bethesda, MD, United States) was used for quantitative analysis of the image (*n* = 4).

### RNA Isolation and Real-Time Quantitative Reverse Transcriptase-Polymerase Chain Reaction

Quantitative reverse transcriptase-polymerase chain reaction (qRT-PCR) was used to detect chondrogenic differentiation-associated gene expression. Cells were seeded and stimulated using the abovementioned methods. After induction culture for 21 days, the medium was discarded, and total RNA was extracted with RNeasy mini kit (Qiagen) according to the instruction manual. The mRNA expression of the cartilage-specific genes (AGC, COL I, COL II, and SOX9) was determined by qRT-PCR using β-actin as an internal reference; the primer sequences are shown in Supplementary Table 1. RT-PCR was performed by reverse transcribing 1 mg of RNA with SuperScript^TM^ III Reverse Transcriptase, followed by PCR with SYBR Premix Ex Taq II (2×) on an ABI 7500 Fast machine (Applied Biosystems, Courtaboeuf, France). Gene expression was calculated using the 2^–ΔΔ*Ct*^ method, where ΔΔCt = (the average value of the gene Ct to be tested – the average value of the reference gene Ct to be tested) – (the average value of the target gene Ct of the control – the average value of the reference gene Ct of the control) (*n* = 5).

### Biochemical Evaluations

All samples were collected and weighed, and then they were tested for total collagen and type II collagen content. Total collagen content was quantified by hydroxyproline assay, and type II collagen content was quantified by sandwich ELISA in accordance with a published method (*n* = 5) (Reddy and Enwemeka, 1996; He et al., 2017, 2018).

### Statistical Analysis

Data analysis was performed using SPSS 25.0 software (SPSS Inc., Chicago, IL, United States). Quantitative data are expressed as mean ± standard deviation (mean ± SD). Data were analyzed by independent-sample *t*-test and one-way analysis of variance. *P* < 0.05 was considered significant.

## Results and Discussion

### Morphological Characterization of Scaffolds

**FIGURE 2:**
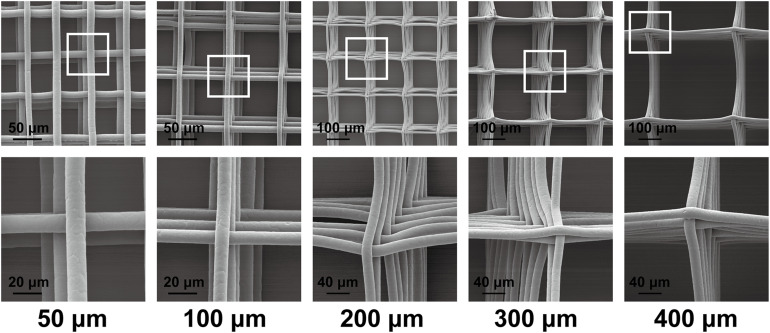
Scanning electron microscopy graphs of the scaffold morphology of 50-, 100-, 200-, 300-, and 400-μm porous scaffolds.

Figure 2 shows the morphology of the scaffolds under a scanning electron microscope. In this study, we prepared a MEW scaffold with high precision. The surface of the printing fiber is smooth, the diameter of the wire is about 10 μm, and the pore size of the scaffold is controllable. These are conducive to follow-up research.

### Comparative Study on Adhesion and Proliferation of Various Cell Types

**FIGURE 3:**
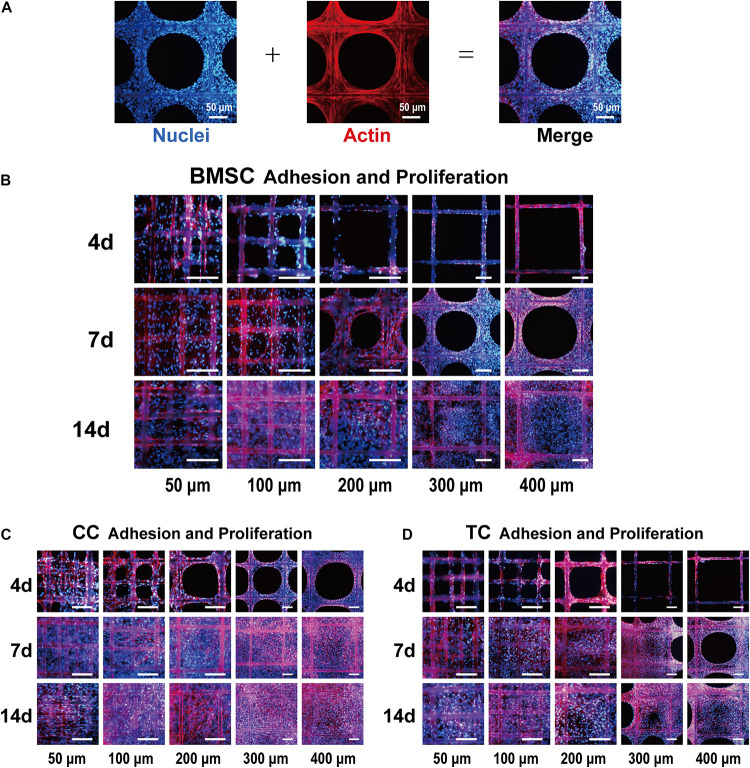
Fluorescence images of bone marrow mesenchymal stem cell (BMSC) adhesion and proliferation. **(A)** Representative example of the cell-loaded scaffold. The left side shows a DAPI (blue)-labeled cell nucleus, the middle shows phalloidin (red)-labeled actin, and the right side shows the merged image. Fluorescence images of BMSCs **(B)**, chondrocytes **(C)**, and tendon cells **(D)** on 50-, 100-, 200-, 300-, and 400-μm porous scaffolds at 4, 7, and 14 days. Scale bar: 100 μm.

Bone marrow mesenchymal stem cells, TCs, and CCs were used in a comparative study of the effect of pore size on cell adhesion and proliferation. For different cells, the most suitable pore size for its adhesion and proliferation might be different. Figure 3A shows the DAPI-labeled nuclei (blue) and the phalloidin-labeled β-actin (red) in BMSCs under a fluorescence microscope and merged images. Figure 3B shows that BMSCs exhibited adhesion and proliferation on 50-, 100-, 200-, 300-, and 400-μm-pore-size scaffolds at 4, 7, and 14 days, respectively. The cell adhesion growth state in each image is significant, indicating that the scaffold has good biocompatibility and is suitable for cell adhesion. At 4 days, the cells in the 50-μm-pore scaffold were almost filled with pores. In the other groups, cell adherence to the surface of each fiber of the scaffold was observed, and there was a tendency to fill the inner pores. At 7 days, the 50- and 100-μm scaffolds were covered and filled by cells; the 200-, 300-, and 400-μm scaffolds also showed a trend of cell ingrowth, which was significantly increased compared with 4 days. At 14 days, the five groups of scaffolds were filled. In each group, compared to 4, 7, and 14 days, the number of cells increased significantly. Among them, the number of cells filled in the 50 μm group increased, and the nuclear field of DAPI significantly increased. Figure 3C shows the CCs’ adhesion and proliferation on 50-, 100-, 200-, 300-, and 400-μm-pore-size scaffolds at 4, 7, and 14 days. Compared to BMSCs at 4 days, chondrocyte adhesion occurred faster, and the number of adherent cells was higher. At 7 days, the chondrocytes spread to fill the 400-μm pores. At 14 days, the number of cells increased slightly compared to that at 7 days. For TCs, Figure 3D shows low cell adhesion and proliferation. Even at 14 days, some of the larger pores were not completely filled. In addition, we found that the cells in the center of the pores are thinner than the cells attached to the fiber due to lack of support. With the completion of filling and further proliferation, the cells and secreted extracellular matrix can gradually fill the entire pore.

**FIGURE 4:**
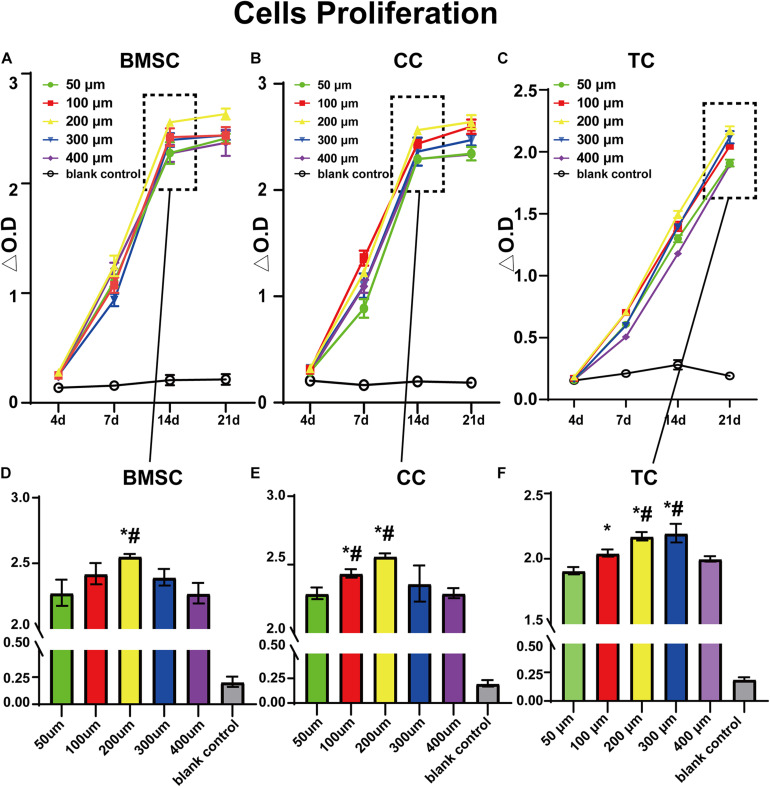
CCK-8 test value for bone marrow mesenchymal stem cells **(A,D)**, chondrocytes **(B,E)**, and tendon cells **(C,F)** in 50-, 100-, 200-, 300-, and 400-μm-pore-size scaffolds at 4, 7, 14, and 21 days. Data represent mean ± standard deviation. *n* = 4; *p < 0.05, compared with the 50 μm groups, ^#^*p* < 0.05, compared with the 400 μm group.

Figure 4 shows the results of the CCK-8 tests at 4, 7, 14, and 21 days after transplanting each group of cells on the scaffolds. First, every group showed an increase in measured values over time, indicating cell adhesion and proliferative activity. Among them, the 200 μm group showed the best result at 14 days for BMSCs. At 21 days, there was only little improvement over the 14 days for BMSCs and CCs. Furthermore, the 200 μm group showed the best result at 21 days, indicating that the environment of the 200-μm scaffold was best for BMSC adhesion and proliferation (Supplementary Figure 1). In the CC group, similar to the BMSC group, the results in each group improved continuously at 4, 7, and 14 days. However, unlike in the case of BMSCs, the 100 μm group showed similar results to the 200 μm group, considering that chondrocytes are small in size and more suitable for small pores. In the TC group, cell viability was lower than that of BMSCs and CCs at 4, 7, and 14 days but similar at 21 days compared to the other two cell types. At 21 days, the TCs showed best results in 300-μm pores, and the results were similar in 200-μm pores, which were only slightly lower than that in 300-μm pores. By contrast, 50 and 400 μm groups had the lowest values, suggesting that the environment of both 300- and 200-μm scaffolds was suitable for TC adhesion and proliferation. Taken together, the comparison of the three cell types demonstrated the different preferences of the cells in terms of pore size. Furthermore, all three cell types showed the lowest value in the 50 and 400 μm groups.

### Comparative Study on the Effect of Pore Size on Chondrogenic Differentiation Using BMSCs

**FIGURE 5:**
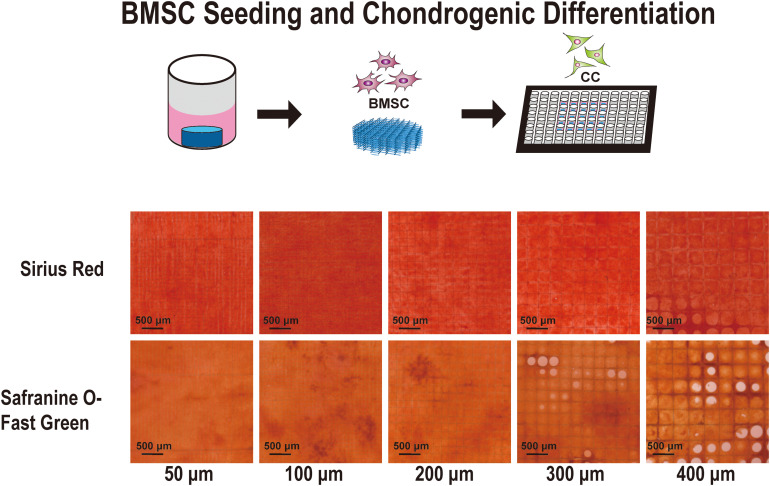
Sirius Red and Safranine O with Fast-green staining of the cell-supported scaffolds. Chondrocyte differentiation from bone marrow mesenchymal stem cells on the 50-, 100-, 200-, 300-, and 400-μm-pore-size scaffolds for 21 days.

To investigate the most appropriate corresponding pore size for cartilage regeneration, BMSCs were implanted on different scaffolds. After cell adherence to the scaffold, the medium was replaced with a chondrogenic differentiation medium. Figure 5 shows the Sirius red and Safranine O-Fast Green staining stereoscopic images of the groups after chondrogenic differentiation on the scaffolds. In the images of the 50, 100, and 200 μm groups, the cells grew well with strong staining patterns. However, in the 300 and 400 μm groups, the cells did not fully grow and fill up all pores. Moreover, it was observed that collagen (Sirius red) and cartilage (Safranine O) in the 100 and 200 μm groups are more prominent.

**FIGURE 6:**
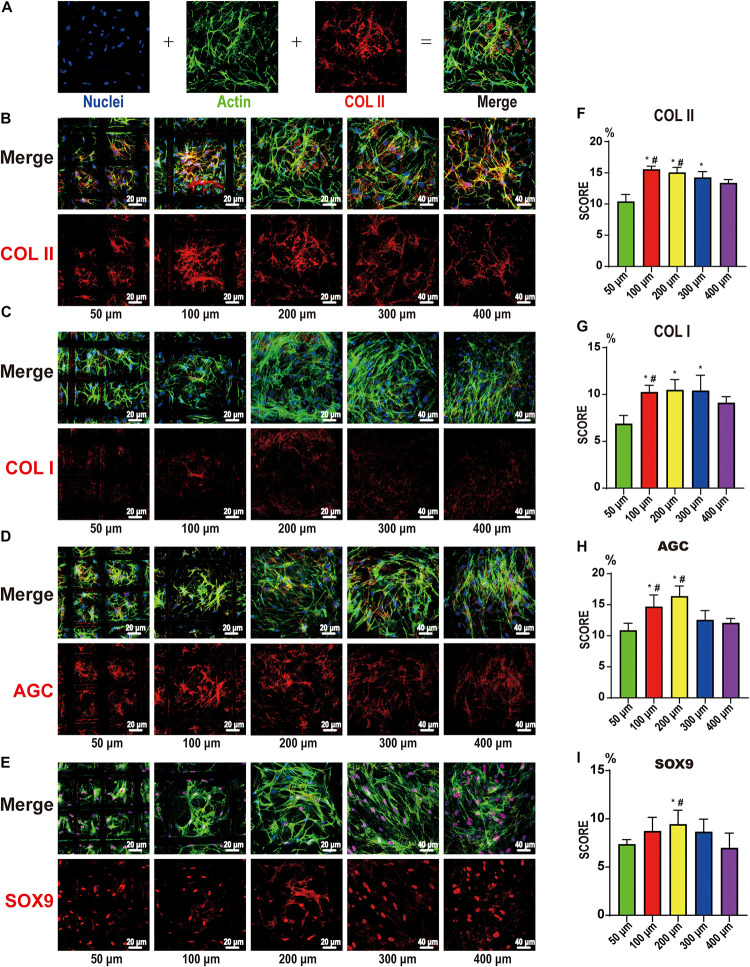
Confocal microscopy for COL I, COL II, AGC, and SOX9. **(A)** Representative example of the confocal microscopy graph of 21-day chondrocyte differentiation from bone marrow mesenchymal stem cells. The first is DAPI (blue)-labeled nuclei, the second is phalloidin (green)-labeled actin, the third is COLII (red), and the fourth is a merged image. COL II **(B)**, COL I **(C)**, AGC **(D)**, and SOX9 **(E)** on the 50-, 100-, 200-, 300-, and 400-μm-pore-size scaffolds. The upper part is the merged image, and the lower part is the protein image (red). Histogram of the results of COL II **(F)**, COL I **(G)**, AGC **(H)**, and SOX9 **(I)** expression in image analysis. Data represent the mean ± standard deviation. *n* = 4; *p < 0.05, compared with the 50 μm groups, ^#^*p* < 0.05, compared with the 400 μm groups.

The expression of COL II, COL I, AGC, and SOX9 after 21 days of stem cell induction is shown in Figure 6. Figure 6A shows the DAPI-labeled nuclei (blue), the phalloidin-labeled β-actin (green), the immunofluorescence for proteins (red), and the merged images. After further analysis, we found that the expression in the 100 and 200 μm groups was the best, especially compared to the 50 and 400 μm groups. This indicates that the pore size of chondrogenic differentiation had a clear range; too large or too small pores are not conducive to differentiation. The 100- and 200-μm pores were more suitable, the 50-μm pore was considered to be too small and oxygen and nutrients cannot be transported, while the 400-um pore is too large, such that the cells proliferate too much, and the differentiation ability was inferior.

**FIGURE 7:**
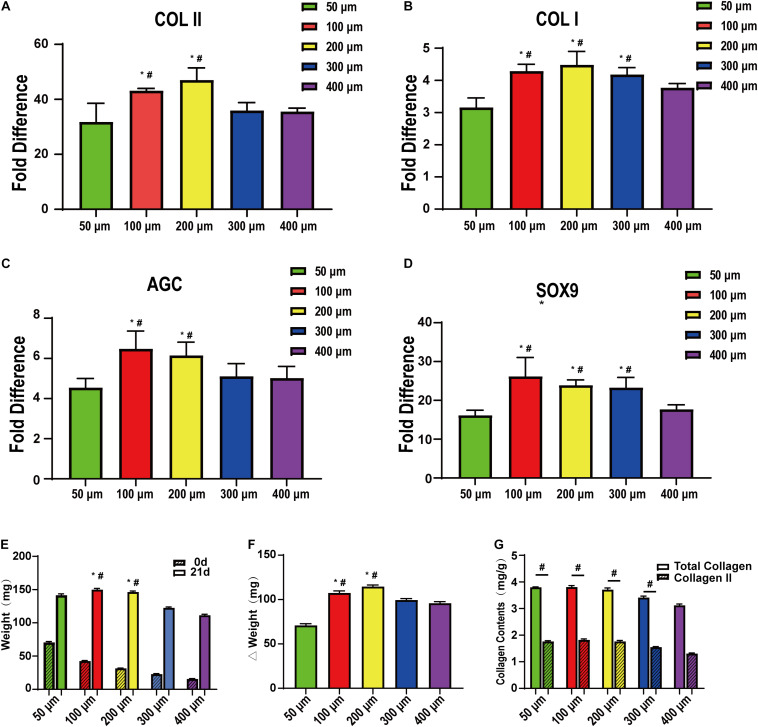
qRT-PCR and quantitative biochemical analysis of bone marrow mesenchymal stem cells on the scaffolds after cartilage induction on 50-, 100-, 200-, 300-, and 400-μm-pore-size scaffolds for 21 days. **(A)** COLII, **(B)** COLI, **(C)** AGC, and **(D)** SOX9 (*n* = 5). **(E)** Simple scaffolds, cell-supported scaffold weight, and **(F)** their gap. **(G)** Total collagen and collagen II content on the scaffolds after cartilage induction on 50-, 100-, 200-, 300-, and 400-μm pore-size scaffolds for 21 days. Data represent the mean ± standard deviation. *n* = 4; *p < 0.05, compared with 50 μm groups, ^#^*p* < 0.05, compared with 400 μm groups.

Figures 7A–D show the mRNA expression of the chondrogenic differentiation marker genes (COL II, COL I, AGC, and SOX9) as determined by qRT-PCR using β-actin as an internal reference. The results were calculated by the 2^–ΔΔ*Ct*^ method. BMSCs were used as a reference, and the value was 1. We further clarified the effect of pore size on chondrogenic differentiation and further confirmed that too large or too small pores are not suitable for cartilage repair. In this part of the study, the 100 and 200 μm groups still showed good expression, while the 50 and 400 μm groups still had lower expression levels. These are consistent with the previous analysis, but, interestingly, in the previous section, the expression of COL II and AGC in the two groups of 100 and 200 μm showed the opposite results. Even if the difference is not obvious, we think that this small difference may be related to the sub-differentiation, such as superficial or mid-deep chondrocytes. Figures 7E–G show the biochemical evaluations after 21 days of stem cell induction. We examined the total weight of each group of scaffolds at 0 and 21 days and finally obtained the weight of the cellular components in the scaffold (Figure 7F). As with the previous studies, the 50 μm group had too small pores and low relative porosity. Oxygen and nutrients cannot be transported well, so cell adhesion and expression were the lowest. The 200 μm group was the highest and higher than the 300 and 400 μm groups. In addition, for the analysis of total collagen and type II collagen content, we found that the collagen content in the 50 μm group was not low, but the total amount was low. The collagen content gradually decreased with the increase of pores, being significantly decreased in the 400 μm group. Therefore, it may be that smaller pores are more capable of promoting collagen production. Thus, we believe that it is more appropriate to use scaffolds with different gradients for cartilage repair. It is more suitable to use smaller pores (such as 100 μm) in the surface layer and larger pores (such as 200 μm) in the middle and deep layers.

### Discussion

Although many studies have investigated the effect of scaffold pore size cells (Nehrer et al., 1997; Tanaka et al., 2010; Pok et al., 2013), due to the limitations of the manufacturing process and accuracy, the overall structure cannot be controlled entirely. With MEW technology, we can accurately prepare 50, 100, 200, 300, and 400-μm-pore-size scaffolds. The microstructure of the scaffold is suitable, and the fiber is smooth. At present, for MEW technology, some researchers have been able to control the fiber diameter to the hundred-nanometer level for stable printing. Although the material requirements are higher than other printing methods, it can provide higher printing accuracy (Hochleitner et al., 2015). Moreover, with the continuous development and application of 3D printing technology, it is vital to further study the effect of 3D printing scaffold microstructure on cells.

In our study, we first verified the impact of the difference in scaffold pore size on cell adhesion and proliferation and that the effect on different cells is different. For cell adhesion, we found in our study that pores that were too small (50 μm) may be more suitable for initial cell adhesion, such that this scaffold could provide more support for the cell. Even with very small pores, cells can easily pass through. During subsequent cell proliferation, cells on the small-pore scaffold were more likely to cross each other and fill the pores (Nuernberger et al., 2011). Therefore, in each group, the 50 μm pores were very easily filled with cells. Later, however, because microscopic pores do not provide enough space, cell proliferation is significantly limited in crowded environments, while slightly larger pores provide a broader environment for cells. However, very large pores take more time for the cells to grow and fill (400 μm) (Youssef et al., 2019). Moreover, it can be noticed that due to the lack of support from the scaffold, the cells in the center of the pores are lower than those on the fiber, and the pores are recessed, but with time, the recesses can be gradually filled.

In addition, the ability of different cells to adhere to the scaffold is different. As previously shown, CCs can climb up the scaffold and fill up the smaller pores more quickly. While it takes longer for larger pores, it still fills reasonably quickly (Bhardwaj et al., 2001; Griffon et al., 2006; Brennan et al., 2019). BMSCs have worse adhesion than CCs, with TCs being the worst. The observed phenomenon is the same as in the previous study, such that cells can easily pass through pores slightly larger than themselves, then slowly climb the scaffold at a faster proliferation rate (Tan et al., 2017). Conversely, studies on cell proliferation have shown that, as we have speculated before, different cells do have their optimal pore sizes, such as CCs were suitable for 100 and 200 μm, BMSCs for 200 μm, and TC for 200 and 300 μm. Thus, our findings provide novel insights for subsequent research, such that we can first explore the appropriate parameters before further repair of different tissues to enhance the repair effect (Salifu et al., 2017; Kankala et al., 2018). Moreover, although we cannot say that all the scaffolds will have the same trend, this similar trend should exist.

After studying the adhesion and proliferation of several cell types on MEW scaffolds with different pore sizes, we investigated the most appropriate corresponding pore size when BMSCs were induced into cartilage. Therefore, BMSCs were implanted on different scaffolds. Following cell adherence to the scaffold, the medium was replaced with a chondrogenic differentiation medium.

In our study, the results showed that the 100 and 200 μm groups were the most suitable for cell chondrogenic differentiation and gene expression. In immunofluorescence and qRT-PCR, the two groups exhibited apparent advantages over the other groups. This was significantly less in the 50 μm group, indicating that the smaller pores (50 μm) were not suitable for chondrogenic differentiation of stem cells.

Moreover, in a more in-depth study of chondrogenic differentiation of BMSCs, we found that microscopic pores were not conducive to cell growth and differentiation. For large pores, i.e., >300 μm, although they provided growth space, excessive cell proliferation also caused a decrease in differential expression. As such, completing tissue repair in a short period using tissue engineering scaffolds is desired, providing enough support to the cells without affecting their function (Gotterbarm et al., 2006; Brunger et al., 2014). Although there were no significant differences between the 100 and 200 μm groups in the study, we still have some interesting findings. In general, both groups could provide sufficient adhesion support for the cells, suitable growth space for the cells, and better performance. However, maybe due to differences in oxygen content or nutrient supply, COL II mRNA expression was higher in the 200 μm group.

Nevertheless, the total amount of COL II is higher in the 100 μm group. This indicates that pore size is also important for cell sub-differentiation. Therefore, in our study, we believe that the 200-μm-pore scaffold may be more suitable for the mid-layer cartilage scaffold, and a 100-μm-pore scaffold may be more suitable for the surface.

Three-dimensional printing technology can precisely control the geometry, mechanical structure, and spatial distribution of the manufacturing structure to mimic the normal structure (Jose et al., 2014; Thomas et al., 2014). Furthermore, different bioactive materials, cytokines, or cells can be loaded at different locations on a structurally controllable basis (Chang et al., 2015; Teimouri et al., 2015; Daly et al., 2016; Xu et al., 2018). Thus, accurate depictions and better replication of the complexity and heterogeneity of endogenous tissues and organs are expected with the construction of a controllable 3D system in the field of regenerative medicine. Therefore, the application of 3D printing technology in tissue engineering provides a new foundation for subsequent development (Lima et al., 2013).

For a tissue engineering scaffold, the gold standard should be its repair ability (Kang et al., 2016; Shi et al., 2017), for which many factors influence the final effect, such as the type of material, biological activity, printed porosity, and pore size, among others (Abdeen et al., 2014; Ryu et al., 2015; Zhu et al., 2018). However, further elucidation of the impact of these factors on the final repair is needed. To build large organizations, a better understanding of the combined effects of pore size on cell adhesion, differentiation, and secretion capacity is required (Chang et al., 2015; Daly et al., 2018; Prasopthum et al., 2018). Our results show that not all cells behave in the same manner on similar scaffold pores, and the difference in scaffold space is also different for the induction effect and protein expression. In the 3D printing process, the pore size can affect the porosity under other conditions unchanged. This correlation is very close, and because the porosity is not intuitive, the adjustment of the pore size is more direct for 3D printing. Thus, controlling the pore size of the scaffold may enhance tissue repair.

There are many shortcomings in this study. Due to the difficulty of standard setting and PCL material as a relatively inert synthetic material, it has yet to be tested *in vivo*. In addition, the experimental divisions (integer of 100, 200, and 300 μm) are not detailed enough; thus, a more detailed stratification may lead to better results. Considering the thickness of the articular cartilage of small animals in subsequent experiments, we only used 200-μm-thick scaffolds. Whether the increase of thickness has an effect on the results still needs further study. Besides this, the number of cell types and the direction of induction are somewhat insufficient. Therefore, to build a more detailed database, efforts by many researchers are needed.

## Conclusion

In this study, 10-layer MEW PCL scaffolds with different pore sizes were prepared, and the effect of pore sizes on cell behavior was studied. First, in the study of the effect of pore size on cell adhesion and proliferation, the rate of adhesion and proliferation of different cells on the scaffold differed, along with the suitable pore size. BMSCs performed best on 200 μm, CCs on 200 and 100 μm, and TCs on 200 and 300 μm. Subsequently, in case of chondrogenic differentiation of BMSCs, pore size had an effect on MSC chondrogenic differentiation, in which 200 μm pore size was more suitable for chondrogenic differentiation. In summary, this study verified that the pore size of 3D-printed, 10-layer MEW PCL scaffolds affected cell adhesion, proliferation, and differentiation.

## Data Availability Statement

The original contributions presented in the study are included in the article/Supplementary Material, further inquiries can be directed to the corresponding author/s.

## Ethics Statement

The animal study was reviewed and approved by 11-20190911 Animal Experimental Ethical Inspection Jiagan Biotchnology Co.

## Author Contributions

YH: conceptualization, methodology, and writing–original draft preparation. ML: methodology, investigation, formal analysis, and validation. QW: investigation, formal analysis, and data curation. ZQ: methodology, investigation, and supervision. BS: writing–reviewing and editing, and supervision. KD: writing–reviewing and editing, supervision, and funding acquisition. All authors contributed to the article and approved the submitted version.

## Conflict of Interest

The authors declare that the research was conducted in the absence of any commercial or financial relationships that could be construed as a potential conflict of interest.
