# Supplementary material for: Effect of Pore Size on Cell Behavior Using Melt Electrowritten Scaffolds
Source: Front Bioeng Biotechnol. 2021 Jul 2;9:629270. doi: 10.3389/fbioe.2021.629270 (PMC8283809; doi:10.3389/fbioe.2021.629270)
Supplement: Supplementary file 1 [file Table_1.docx]

**Table S1.** Forward and reverse primers used for quantitative RT-PCR

| Gene | Primer sequence | Product length (bp) | Species |
| --- | --- | --- | --- |
| COL II | F:5′-CCACGCTCAAGTCCCTCAACAA-3′  R:5′-TCCAGTAGTCACCGCTCTTCCA-3′ | 129 | Oryctolagus |
| COL I | F:5′-AAGCCGGTCGTGATGGCAA-3′  R:5′-CACCGACGGGACCAATAGAACC-3′ | 207 | Oryctolagus |
| AGC | F:5′-TTGGAGGTCGTGGTGAAAGG-3′  R:5′-TGGGGTACCTGACAGTCTGA-3′ | 145 | Oryctolagus |
| SOX9 | F:5′-AAGATGACCGACGAGCAGGAGA-3′  R:5′-TGTTCTTGCTGGAGCCGTTGAC-3′ | 280 | Oryctolagus |
|  | F:5′-TCTTCCAGCCCTCCTTCCTG-3′  R:5′-CGTTTCTGCGCCGTTAGGT-3′ | 142 | Oryctolagus |

F: forward; R: reverse; COLII: collagen type II; COL I: collagen type I; AGC: aggrecan; SOX9: sox-9


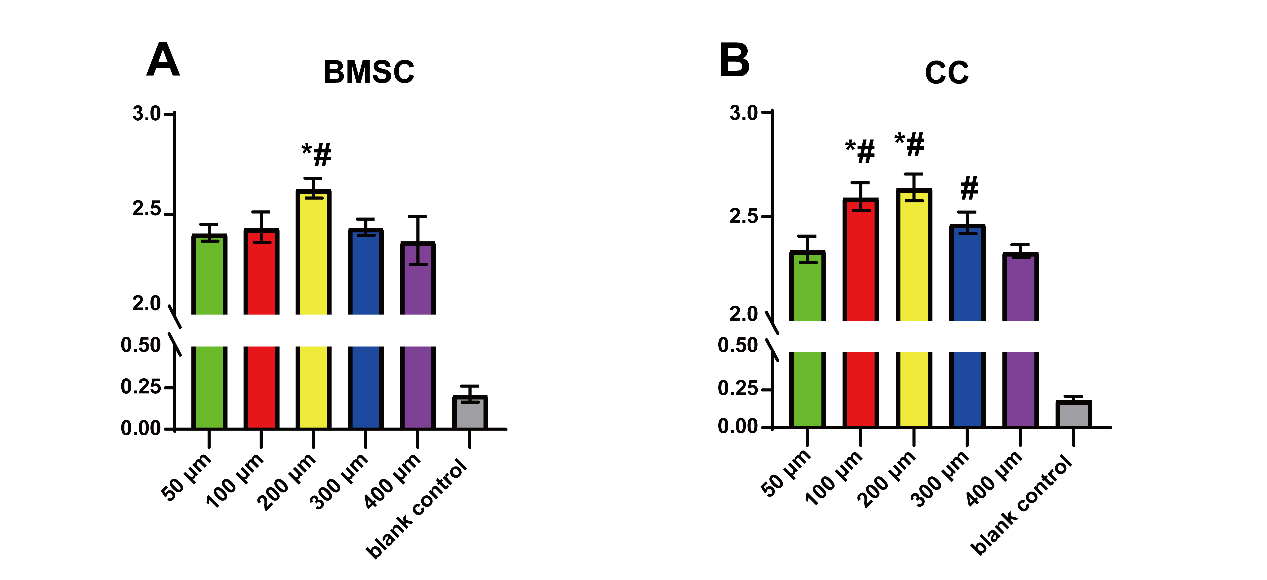


Figure S1. CCK-8 test value for BMSCs (A) and CCs (B) in 50, 100, 200, 300, and 400 μm pore size scaffolds at 21d. Data represent the mean ± standard deviation. (n=4) (*p < 0.05, compared with 50 μm groups，#p<0.05, compared with 400 μm group).


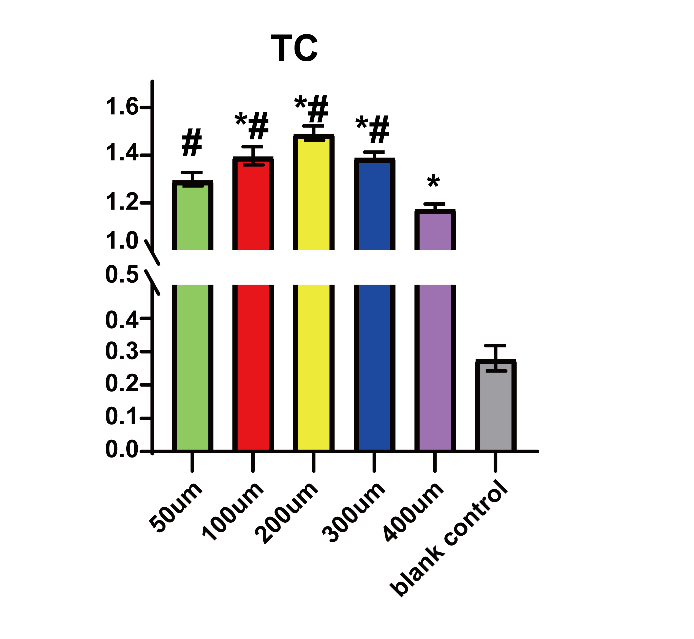


Figure S1. CCK-8 test value for TCs（TC） in 50, 100, 200, 300, and 400 μm pore size scaffolds at 14d. Data represent the mean ± standard deviation. (n=4) (*p < 0.05, compared with 50 μm groups，#p<0.05, compared with 400 μm group).
